# A step-down photophobic response in coral larvae: implications for the light-dependent distribution of the common reef coral, *Acropora tenuis*

**DOI:** 10.1038/s41598-020-74649-x

**Published:** 2020-10-19

**Authors:** Yusuke Sakai, Kagayaki Kato, Hiroshi Koyama, Alyson Kuba, Hiroki Takahashi, Toshihiko Fujimori, Masayuki Hatta, Andrew P. Negri, Andrew H. Baird, Naoto Ueno

**Affiliations:** 1 Division of Morphogenesis, National Institute for Basic Biology, Okazaki, Aichi Japan; 2 Exploratory Research Center on Life and Living Systems (ExCELLS), National Institutes of Natural Sciences, Okazaki, Aichi Japan; 3 Department of Imaging Science, Center for Novel Science Initiatives, National Institute for Basic Biology, Okazaki, Aichi Japan; 4 Division of Evolutionary Biology Biodiversity, National Institute for Basic Biology, Okazaki, Aichi Japan; 5 Division of Embryology, National Institute for Basic Biology, Okazaki, Aichi Japan; 6 Department of Basic Biology, School of Life Science, SOKENDAI (The Graduate University for Advanced Studies), Okazaki, Aichi Japan; 7 ARC Centre of Excellence for Coral Reef Studies, James Cook University, Townsville, QLD Australia; 8 Department of Biology, Ochanomizu University, Bunkyo-ku, Tokyo, Japan; 9 Australian Institute of Marine Science, Townsville, QLD Australia; 10 Present Address: Department of Biology and Geosciences, Graduate School of Science, Osaka City University, Sumiyoshi-ku, Osaka, Japan

**Keywords:** Behavioural ecology, Ecology, Zoology

## Abstract

Behavioral responses to environmental factors at the planktonic larval stage can have a crucial influence on habitat selection and therefore adult distributions in many benthic organisms. Reef-building corals show strong patterns of zonation across depth or underwater topography, with different suites of species aggregating in different light environments. One potential mechanism driving this pattern is the response of free-swimming larvae to light. However, there is little experimental support for this hypothesis; in particular, there are few direct and quantitative observations of larval behavior in response to light. Here, we analyzed the swimming behavior of larvae of the common reef coral *Acropora tenuis* under various light conditions. Larvae exhibited a step-down photophobic response, i.e. a marked decrease in swimming speed, in response to a rapid attenuation (step-down) of light intensity. Observations of larvae under different wavelengths indicated that only the loss of blue light (wavelengths between 400 and 500 nm) produced a significant response. Mathematical simulations of this step-down photophobic response indicate that larvae will aggregate in the lighter areas of two-dimensional large rectangular fields. These results suggest that the step-down photophobic response of coral larvae may play an important role in determining where larval settle on the reef.

## Introduction

The mechanism underlying the formation of species-specific distribution patterns is a fundamental question to be solved in ecology. Many benthic marine organisms have a planktonic larval stage and a sessile adult stage, and behaviors of the planktonic larvae can be a primary determinant for the distribution of adults^1–3^. Accordingly, understanding how larvae sense and change their behavior in response to external stimuli can help to identify environmental factors that influence larval dispersal and settlement.

Adult reef-building scleractinian corals are sessile and rely on the production of planktonic planula larvae to aid in dispersal. Although numerous environmental factors can affect the survival and distribution of adult corals, light is considered one of the primary factors because corals are largely dependent on photosynthate produced by endosymbiotic dinoflagellate algae to meet their metabolic demands. As a result, the habitat of reef-building corals is mostly limited to the euphotic zone, where the light intensity exceeds 1% of its sea surface light level^4,5^. However, within the euphotic zone, most coral species have highly specific distribution patterns across depth and among the many complex microenvironments on reefs^6,7^, one of the main causes of which is likely to be predictable variation in light conditions.

As in many other benthic marine organisms, habitat selection at the time of larval settlement strongly affects the distribution of adult corals^8,9^. Larval swimming and settlement behaviors are affected by many factors. Larvae tend to aggregate in light or dark regions in experimental chambers with a light gradient and the strength and direction vary considerably with species, age, temperature, and the intensity and wavelength of light^10–14^. Kawaguti (1941) first suggested that differences in the behavioral light response among larvae of different species could explain difference in the depth distribution of adults^10^. In addition, the settlement site of coral larvae can be affected by light intensity, spectral quality and the colour of the substratum^15–19^.

The behavioral response of motile microorganisms to light can be categorized in three ways^20,21^. Phototaxis is defined as movement in the direction of a light source. Photokinesis describes the steady state velocity of organisms according to the absolute intensity of light stimuli. A photophobic response is defined as the transient alteration of swimming activity in response to a sudden attenuation (step-down) or amplification (step-up) of stimulus light intensity^20,21^. These elementary light responses could be one of the determinants for the light-dependent distribution of corals. Previous studies have described light-dependent behaviors observed in coral larvae as phototaxis. For example, Kawaguti (1945) reported that coral larvae showed positive or negative phototaxis depending on the distance from the light source and further hypothesized that this behavior influenced the ecological distribution of species on the reef^10^. However, Kawaguti’s (1945) preliminary experiments did not prove that larvae exhibit phototaxis in a strict sense. To investigate the precise characteristics of the immediate response to variable light stimuli, real-time observations and quantitative analyses of larval swimming behavior under different light conditions are required.

In this study, we observed and analyzed the swimming behavior of larvae of the reef-building coral, *Acropora tenuis* (Dana, 1846), in response to an attenuation of stimulus light. We next tested the sensitivity of larvae to wavelengths of light between 320 and 680 nm. Finally, we developed a mathematical model to test whether the observed light response resulted in aggregation or dispersal under specific light fields and discuss the potential consequences for the distribution of adults on the reef.

## Results

### Experiment 1—larval response to a rapid attenuation of stimulus light

**Figure 1:**
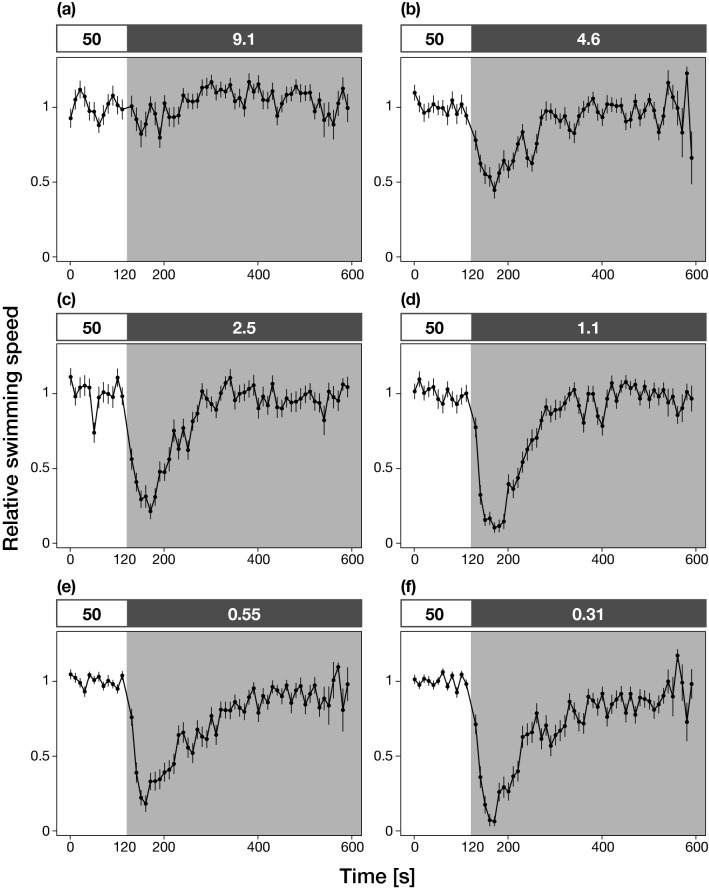
Photophobic response in *Acropora tenuis* larvae induced by the rapid attenuation of stimulus white light. Each graph shows time series plots of the relative swimming speed before and after the rapid attenuation of the stimulus light. The relative swimming speed was defined as the swimming speed of each individual divided by the mean swimming speed before the attenuation of the stimulus light calculated for each movie. The solid circles and bars indicate the means ± SEs (n = 10–15 larvae). The boxes with numbers on the top of each graph represent the photon flux densities before (< 120 s, 50 µmol/m^2^/s) and after (> 120 s, represented by the darker shaded box, (**a**) 9.1, (**b**) 4.6, (**c**) 2.5, (**d**) 1.1, (**e**) 0.55, and (**f**) 0.31 µmol/m^2^/s, respectively) the light attenuations. All graphs shown were produced using the *ggplot2* function in the R version 3.5.1 and edited by Adobe Illustrator CS6 (Adobe, San Jose, California, USA).

**Figure 2:**
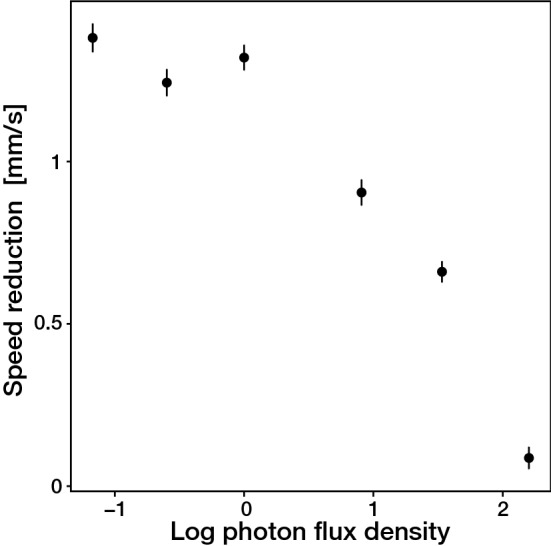
Relationship between the degree of the light attenuation and swimming speed reduction. The absolute degree of swimming speed reduction before and after the light attenuation is plotted against the light intensity after the light attenuation. The light intensity is represented as the logarithmic transformed photon flux density (µmol/m^2^/s). The swimming speed reduction was the differences between the swimming speed before (for 0–120 s in the Fig. 1) and right after (for 150–250 s in the Fig. 1) the light attenuation. Solid circles with error bars represent means ± SEs (n = 10–15 larvae). The plot was produced using the *ggplot2* function in the R version 3.5.1 and edited by Adobe Illustrator CS6 (Adobe).

To examine whether *Acropora tenuis* larvae respond to rapid changes in light levels, we observed and recorded the larval swimming activity under various light regimes. *A. tenuis* larvae exhibited helical swimming behaviors using cilia (Supplementary Movies S1 and S2), and the mean swimming speed ± standard deviation under illumination of constant white light with 50 µmol/m^2^/s was 1.96 ± 0.80 mm/s. Figure 1 shows the time series plots of the relative swimming speeds before and after the rapid attenuations of stimulus white light. Ten-min observations of larval swimming behaviors revealed that the larvae decreased swimming activity 10 s after the intensity step-down and the mean swimming speed reached a minimum 30–40 s after the intensity step-down, after which larvae resumed swimming at the initial speed (Fig. 1; Supplementary Movie S3). The observed swimming behavior indicated that *A. tenuis* larvae had a step-down photophobic response. The reduction in swimming speed was correlated with the degree of the light attenuation such that switching to the lower light levels caused the strongest responses (larger speed reduction) (Fig. 2). We found no directed movement and no difference in the swimming speed with respect to the direction of the light source (Supplementary Fig. S1 and S2).

### Experiment 2—larval response to changes in the wavelength of stimulus light

**Figure 3:**
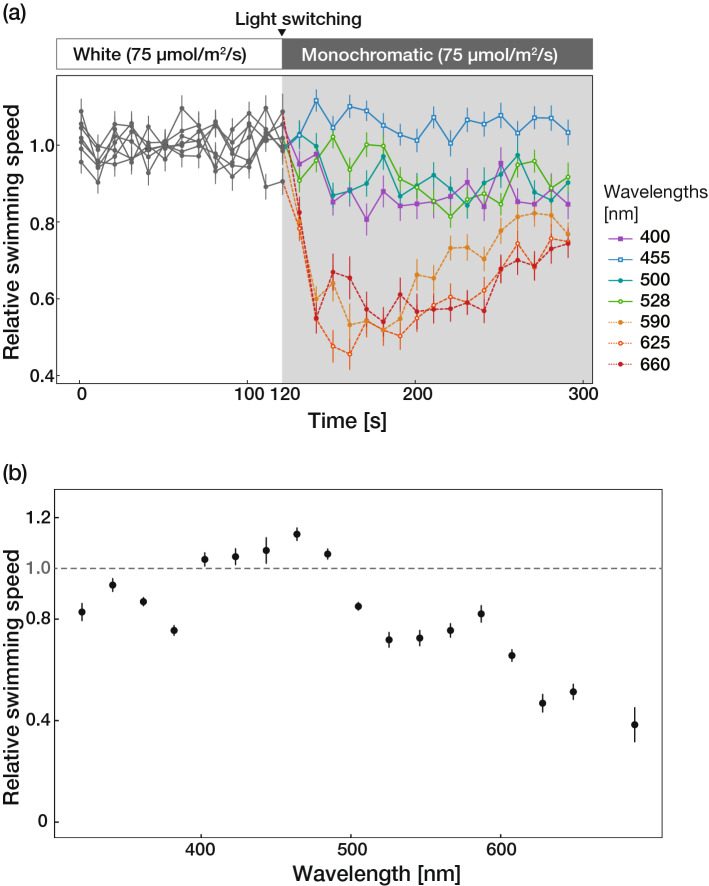
Photophobic response in *Acropora tenuis* larvae induced by the rapid change in wavelengths of stimulus light. (**a**) A time series plot of relative swimming speed of *Acropora tenuis* larvae before and after the rapid change in the wavelengths of stimulus light. The relative swimming speed was defined as the swimming speed of each individual divided by the mean swimming speed under white LED light stimulation. The upper schematic represents the light regime of the experiment. Each point shows the means ± SEs (n = 10–15 larvae). (**b**) The relative swimming speeds measured between 30 to 90 s after switching are plotted against wavelengths. Solid circles with error bars indicate means ± SEs (n = 10–15 larvae). The figures a and b were produced using the *ggplot2* function in the R version 3.5.1 and edited by Adobe Illustrator CS6 (Adobe).

A photophobic response was also elicited by rapid changes in the wavelength of stimulus light. The larvae transiently ceased their swimming behavior in response to switching from white light to long wavelengths of light (590, 625, and 660 nm), while no clear response was observed when white light was switched to short wavelengths of light (400, 455, 500 and 528 nm; Fig. 3a; Supplementary Movies S4 and S5).

To more precisely test the wavelength sensitivity of *A. tenuis* larvae, we observed the photophobic response when white light was switched to 18 different monochromatic light stimuli. Figure 3b shows the relative swimming speeds from 30 to 90 s after switching illumination from white light to each monochromatic light stimulus plotted against the wavelengths of light. The relative swimming speeds of larvae in < 400 nm and ≥ 500 nm light were lower than 1, indicating that larvae had a photophobic response when moving from white light to each of monochromatic lights with wavelengths in these ranges. In particular, almost all larvae had a photophobic response when the white light was switched to light of wavelengths above 600 nm such that the mean relative swimming speeds after switching were reduced to 0.4–0.5 mm/s. In contrast, larvae showed no response (no clear swimming speed reduction) when the white light was switched to monochromatic lights between 400 and 500 nm (Fig. 3b; Supplementary Fig. S3). These results indicate that the depletion of light in the blue wavelengths (400–500 nm) was the key for the step-down photophobic response.

### Simulation of larval accumulation in light–dark field

**Figure 4:**
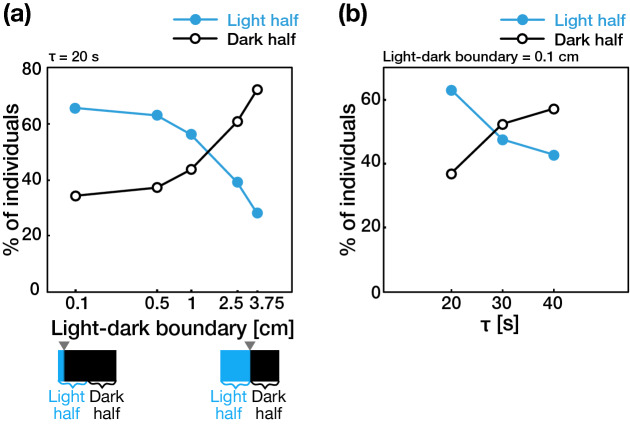
Results of the mathematical simulation of larvae having a step-down photophobic response in a “small” rectangular field (7.5 × 2.5 cm). The proportion of larvae accumulated in light and dark half of a “small” rectangular area are plotted against (**a**) position of the light–dark boundary and (**b**) time required to stop swimming. The time required to stop swimming (seconds) was the value with the mean τ ± 50% fluctuation given by a uniform distribution. The boxes in the bottom of the figure a represent the schematics of rectangular areas in the simulations and black arrows indicate the light dark boundaries. See also Supplementary Fig. S4 and “Methods” section for the details of the model. The graphs shown were produced using Microsoft Excel for Mac ver. 16.41 (Microsoft, Redmond, Washington, USA) and edited Adobe Illustrator CS6 (Adobe).

**Figure 5:**
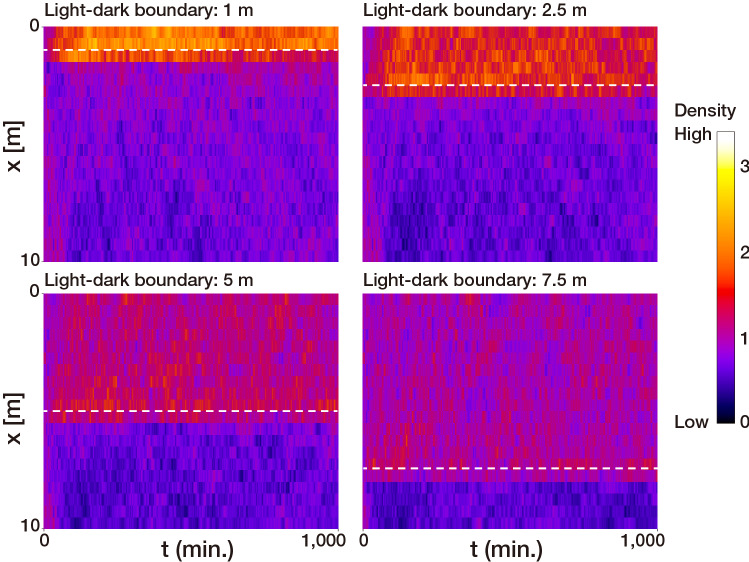
Results of the mathematical simulation of larvae having a step-down photophobic response in a “large” rectangular field (10 × 0.1 m). Figures show densities of the larvae along x-axis (x = 0: light side; x = 10: dark side) from 0 to 1000 min. The densities are standardized to a mean density of 1, represented as color brightness, and shown at 0.5 m × 2 min resolution in the figures. White dashed lines represent the positions of the light–dark boundaries. The time required to stop swimming was set to 20 ± 10 s (τ = 20) in the all simulations shown. ImageJ software was used for visualizing the density values, and the figures were edited by Adobe Illustrator CS6 (Adobe).

We mathematically tested whether the step-down photophobic response observed in *A. tenuis* larvae can cause a specific distribution pattern in a two-dimensional region divided into light and dark areas with a sharp light–dark boundary by performing computational simulations (see “Methods” section and Supplementary Fig. S4a, b for details). First, to test whether the step-down photophobic response causes a biased distribution pattern in a small-enclosed area, we ran the simulation in a two-dimensional “small” rectangular field (7.5 × 2.5 cm) of a size corresponding to the size of the chamber used in earlier research that reported putative phototaxis in coral larvae^10^. In the “small” rectangular field, the modeled larvae having a step-down photophobic response accumulated in either the light half (0 cm ≤ x ≤ 3.75 cm) or the dark half (3.75 cm ≤ x ≤ 7.5 cm) of the area depending on two parameters: the position of light–dark boundary and the time required to stop swimming. The number of larvae was higher in the light half when the position of the light–dark boundary shifted to the light side (i.e. when the dark region was larger than the light region), whereas the number of larvae in the dark half increased with the movement of the light–dark boundary from the light half to the dark half (Fig. 4a). Also, larvae accumulated in the light half when the time required to stop swimming was set to 20 ± 10 s (τ = 20) i.e. consistent with the actual time required to stop swimming reported in experiment 1 (Fig. 1; Supplementary Fig. S4c), while larvae accumulated in the dark half when the time was longer than 30 ± 15 s (τ = 30 and τ = 40, Fig. 4b). Next, to estimate the distribution patterns of larvae that exhibit a step-down photophobic response in large open water environments, we ran the simulation in metre-scale “large” rectangular fields (1.5, 3, 5, 10, 20, 30, and 100 × 0.1 m). Accordingly, in all the “large” rectangular fields simulated, the density was higher on the lighter side of the light dark boundary than in the darker side regardless of the position of the light–dark boundaries (Fig. 5; Supplementary Fig. S5). Our mathematical model and simulations raised the possibility that the photophobic response of larvae can drive their biased distribution in the natural environment depending on light.

### Candidate photoreceptors in the genome of *Acropora tenuis*

To identify the candidate photoreceptors which could drive the step-down photophobic response, we performed a similarity search against the *A. tenuis* genome using previously reported opsin and photolyase/cryptochrome protein sequences as the query. We found seventeen opsin genes having conserved retinal binding sites on the *A. tenuis* genome, which were categorized into three cnidarian opsin families (Supplementary Fig. S6). Moreover, *A. tenuis* has 4 photolyase/cryptochrome genes with the N-terminal Photolyase-Homologous Region (PHR) domain that binds to the chromophore flavin adenine dinucleotide (Supplementary Fig. S7).

## Discussion

In the present study, *Acropora tenuis* larvae showed a strong step-down photophobic response, and mathematical simulations confirmed that this response causes biased distribution patterns according to light environments. Although several previous studies have reported the larval light response in corals, these studies mostly focused on measuring the settlement frequency or the accumulation rate after long exposures to two or three different light stimuli^16,18^. The present study included real-time tracking of larval swimming behavior and elucidated the immediate response to temporary changes in light stimuli at the individual level. Moreover, we investigated the wavelength sensitivity of *A. tenuis* larvae by observing the behavioral response to 18 different monochromatic light stimuli and found that the larvae showed the photophobic response when switching from white light to UV or long-wavelength light, while there was no response when switching to blue (between 400 and 500 nm) light.

We found no biased movement toward the light source (i.e. the larvae did not exhibit “phototaxis” in a strict sense^21^), but our simulation results indicated that the step-down photophobic response resulted in larvae accumulating in either the light side or the dark side in a “small” enclosed rectangular field (7.5 cm × 2.5 cm) equivalent to the size of experimental chamber used in the phototaxis assays in Kawaguti (1945)^10^. Kawaguti (1945) reported that coral larvae showed positive or no phototaxis under illumination with low light intensity, but the phototactic response became negative under high light intensity^10^. The position of the light–dark boundary can be interpreted as representing the position where the light intensity is equal to the larval detection threshold of light intensity. Under the high light conditions, this position would move away from the light source, resulting in the shift of the boundary to the dark side. Based on this assumption, the larval behavior observed in Kawaguti (1945) is consistent with the results of our simulation, which showed that the position of the light–dark boundary affected larval distribution: larvae accumulated in the light half when the area of the light region was small (corresponding to low light intensity conditions), while the number of larvae in the dark half increased with the expansion of the light region (corresponding to the increase in light intensity)^10^. In addition, the time required to stop swimming also affected the area in which larvae accumulate. Thus, these biased distribution patterns in the small-enclosed experimental chamber caused by the step-down photophobic response of larvae likely underlie the reported difference in the strength or sign (positive or negative) of phototaxis in larvae with age^11,13^, temperature^12^, and species^10^.

In contrast to the results observed in the “small” enclosed fields, in metre-scale “large” rectangular fields where the effect of the outer boundary (in a small area, larvae frequently bounce off the wall of the chamber, affecting the final distribution) can be ignored, the larvae exhibiting a step-down photophobic response clearly accumulated in the light side, keeping a sharp border at a position slightly beyond the dark side of the light–dark boundary. This result suggests that the step-down photophobic response will cause larvae to aggregate in the light region in open waters or just beyond light–dark boundaries on reefs. A step-down photophobic response has previously been described in photosynthetic microorganisms such as *Chlamydomonas*^22,23^ and *Euglena*^24–26^, where it facilitates the accumulation of microalgae in light regions suitable for photosynthesis. Similarly, a step-down photophobic response observed in the present study could guide coral larvae to settle at sites on the reef that have suitable light conditions for the photosymbiotic adults.

The actual distribution of larvae in a three-dimensional natural open water setting is determined by complicated interactions of behavioral and physiological characteristics of larvae. Most coral eggs and developing larvae are positively buoyant and often aggregate on the ocean surface following spawning^27,28^. However, despite being poor swimmers^29^, these aggregations break up once larvae develop motility. Once motile, larvae swim towards and explore the substratum where metamorphosis is influence by diverse cues such as biogenic chemicals from bacteria and algae. A step-down photophobic response might influence the settlement site in a number or ways. In the pelagic stage, a step-down photophobic response to the attenuation of light with depth will cause *A. tenuis* larvae to accumulate in the lighter, shallow areas of the reef. Once larvae have been entrained on the reef, they will experience complex variations in light intensity caused by structural features such as established coral colonies, spurs, groves, ledges and micro-crevices. These underwater light conditions will elicit the step-down photophobic response of larvae at light–dark boundaries, leading to skewed population density of larvae according to light conditions. Thus, the step-down photophobic response could be one of the elementary responses that determine the final settlement location by broadly influencing the light-dependent distribution patterns of larvae in the natural open water environment.

The switching from white light (i.e. a combination of lights with different wavelengths in the visible spectrum) to monochromatic light with wavelengths below 400 nm and above 500 nm induced the photophobic response (Supplementary Fig. S3), indicating that switching to those wavelengths of light was equivalent to the reduction in white light intensity for the sensory apparatus of *A. tenuis* larvae. This suggests that the larvae have high detection sensitivity to wavelength of light between 400 and 500 nm, and low sensitivity to < 400 nm and > 500 nm light. In particular, *A. tenuis* larvae have little or no sensitivity to long wavelengths (> 600 nm) of light (Supplementary Fig. S3). In natural seawater, UV light and long wavelengths of light rapidly attenuate with depth, while blue light penetrates much deeper^30^. Therefore, the larvae are likely to use blue light as a reliable gauge of the light gradient along depth or underwater topography to regulate their swimming behavior.

The pattern of wavelength sensitivity suggests that single or multiple blue light receptors are involved in the step-down photophobic response in *A. tenuis* larvae. There is some evidence for photoreceptors, including rhodopsins and cryptochromes, in corals of the genus *Acropora*^31,32^. For example, opsin proteins are expressed in larvae of *A. palmata*, some of which activated G proteins, suggesting that an opsin based phototransduction cascade might operate in these larvae^32^. In the *A. tenuis* genome, there are seventeen opsin genes and 4 photolyase/cryptochrome genes with the N-terminal Photolyase-Homologous Region (PHR) domain. Any of these proteins could function as a photoreceptor and be involved in regulating ciliary motion, leading to the step-down photophobic response in larvae. Larvae of *A. tenuis* do not contain zooxanthellae and consequently, the light response is likely due to photoreception of the larvae. However, in larvae that do have zooxanthellae, the light response might also be affected by photoreception of the symbionts. Furthermore, during swimming and settlement, coral larvae experience multiple external stimuli other than light, such as water currents, the texture of the substratum and chemical signals^33^, and thus these other sensory inputs are also likely to influences larval behavior. Further analyses are required to fully understand the relationship between these sensory modalities and behaviors in coral larvae that influence settlement location in natural environments.

## Methods

### Experiment 1—larval response to a rapid attenuation of stimulus light

*Acropora tenuis* (Dana, 1846) is a common reef-building scleractinian coral in shallow water habitats throughout the Indo-Pacific Ocean. Seven adult colonies of *A. tenuis* were collected at 2–5 m depth from Backnumbers Reef (S18°29.26′, E147°09.18′), a mid-shelf reef in the central Great Barrier Reef (GBR) in November 2018, and transferred in flow-through tanks over 8 h by ship to flow-through aquaria in the National Sea Simulator (SeaSim), Australian Institute of Marine Science (AIMS), Queensland, Australia. This facility uses natural coastal seawater filtered to 1 µm and the range of water quality parameters matched that of mid-shelf reefs including Backnumbers Reef in November: temperature 26.5–27.5 °C, salinity 36.4–36.5 psu and pH 8.13–8.17. Immediately after spawning on November 5, gamete bundles were mixed to fertilize eggs, and cultures of embryos then larvae were maintained in 500 L flow through seawater tanks (0.5 µm filtered), with the motile aposymbiotic planula larvae becoming competent to settle four days after the spawning. Five to nine day old larvae were used in this experiment, with 10–15 larvae transferred into a rectangle polystyrene chamber (6.5 cm × 3.5 cm × 1 cm) filled with 15 mL 0.5 µm-filtered seawater (FSW). To examine whether larvae respond to rapid changes in the photon flux density of stimulus light, the swimming behavior of larvae was observed under the following light scheme. Firstly, a single long side of the test chamber was illuminated for 120 s using a 50 µmol/m^2^/s white LED light (ISC-201-2; CCS Inc., Kyoto, Japan), and the normal swimming activity of the larvae was recorded. Subsequently, the stimulus light was rapidly (< 1 s) attenuated using neutral density filters (No. 209, 210, 211, 298 and 299; Lee Filters, Andover, UK) such that the same white light illuminated the test chamber but with various photon flux densities. The five neutral density filters produced six different light stimuli each with a different photon flux density (9.1, 4.6, 2.5, 1.1, 0.55, 0.31 µmol/m^2^/s), all measured at the center of the experimental chamber using a Jaz spectrometer (Jaz-EL200; Ocean Optics, Dunedin, FL, USA). The swimming behavior of the larvae during the light exposure was recorded from above with a NIKON 1 J5 digital camera equipped with a NIKON Ai AF MICRO-Nikko 60 mm macro lens (NIKON, Tokyo, Japan). To avoid any potential effect of diurnal variation in the swimming activity on the results, all observations were recorded between 9:00 and 11:00, and the temperature was maintained at 26 °C.

### Experiment 2—larval response to changes in the wavelength of stimulus light

Adult colonies of *A. tenuis* were collected at 1–3 m depth before the predicted spawning day in November 2017 from Little Pioneer Bay, Orpheus Island, GBR (S18°36.35′, E146°29.14′) and were placed into out-door raceways with constant flow-through seawater at the Orpheus Island Research Station (James Cook University). Spawning occurred on 8 November, 2017 and fertilization and larval culturing followed the methods of Experiment 1, however, the planula were maintained in plastic buckets (15 L) in 0.5 µm filtered seawater which was changed daily. 10–15 larvae aged 7–9 days were transferred into the test chamber as previously described. We first illuminated the larvae with 75 µmol/m^2^/s white LED light for 2 min and then the white light was replaced with one of each quasi-monochromatic light stimuli (400-, 455-, 500-, 528-, 590-, 625- or 660-nm light) with the same photon flux density as the white light (75 µmol/m^2^/s) for 3 min each. Different wavelengths of light were produced using eight different colours of HOLOLIGHT (Pi PHOTONICS, Inc., Hamamatsu, Japan) connected to DMX light controller. Larval swimming behaviors were again recorded from above.

To investigate the wavelength sensitivity of the larvae at higher spectral resolution, we observed the swimming behavior under monochromatic light stimuli in increments of 20 nm between 320 and 680 nm using the Okazaki Large Spectrograph (OLS) at the National Institute for Basic Biology (NIBB), Okazaki, Japan^34^. For this experiment, we collected colonies of *A. tenuis* from < 3 m depth on the fringing reef on Sesoko Island (N26°37.58′, E127°52.01′) and kept them in flow-through aquaria at Sesoko Station (Tropical Biosphere Research Center, University of the Ryukyus, Okinawa, Japan). Four days after spawning, motile planula larvae were transported to NIBB following the method described previously^35^. Briefly, larvae were transferred into 1 L plastic bottles at a density of 3000 individuals/L and then shipped by express service or as hand luggage. The transport duration did not exceed 2 days. The survival of coral larvae was > 90% with this transportation procedure. After arrival, larvae were transferred to plastic bowls with transport water, and then gradually acclimated to artificial seawater (LIVESea Salt, DELPHIS, Hyogo, Japan) at 26 °C. These larvae were kept at a density of approximately 1000 individuals/L and seawater was exchanged daily. 10–15 larvae in the test chamber were illuminated by 35 µmol/m^2^/s white LED light for two minutes and then the white light was replaced with each of monochromatic light stimuli (320–680 nm) with the same photon flux density (35 µmol/m^2^/s) produced by OLS from a single long side of the test chamber. Swimming tracks were recorded from above. The photon flux densities were measured at the center of the experimental chamber using Light Analyzer LA-105 (Nippon Medical and Chemical Instrument CO., Osaka, Japan). The difference in the photon flux densities between experiments (50, 75 and 35 µmol/m^2^/s, respectively) resulted from the difference in light apparatus used among experiments. In Experiment 2, to equalize the photon number of each light stimulus with different wavelengths, the photon flux density of all light stimuli was set to match that of a wavelength of light with the weakest photon flux density. The photon flux density values of stimulus light used in this study (< 100 µmol/m^2^/s) were within the range of those measured in cryptic habitats like shaded areas or vertical/downward-facing horizontal surfaces of substrata in shallow—middle waters (< 10 m depth)^15,36^, where *Acropora tenuis* larvae are likely to settle^7,16^.

### Tracking analysis of recorded larval swimming

All movie files from Experiment 1 and 2 were processed with in-house applications written in Objective-C, C and Ruby, and the swimming speeds of the larvae were calculated using R version 3.5.1^37^. First, we extracted movie frames and converted them to a sequence of still images at 20 frames per second; this frame rate value was adopted so that identical larvae mostly overlapped between two successive movie frames. The movie frames were not extracted from full movies because of the large data size, instead, we clipped two-seconds-long movie fragments (40 frames each) every 10 s from each full movie. Next, for each 2 s-long movie fragment, we highlighted the larval silhouettes by the background subtraction and then used global thresholding to extract larval bodies. Trajectories of identical larvae were produced by frame-by-frame tracking based on nearest neighbor algorithm. Subsequently, we selected proper trajectories of larvae in each movie fragment based on their size and roundness. We calculated the mean swimming speed of each larval trajectory in 2 s-long movie fragments by measuring the distance travelled between two successive movie frames divided by 0.05 s. We also calculated the “relative swimming speed”, defined as the swimming speed divided by the mean swimming speed before switching the light stimuli (i.e. divided by the mean swimming speed from 1 to 120 s in each movie).

### Mathematical modeling and simulation

We constructed a mathematical model for larvae exhibiting a step-down photophobic response and computed the spatial distribution of the “modeled larvae” in two-dimensional chambers (Supplementary Fig. S4). Each individual larva was modeled as a self-propelled particle moving a constant velocity (2.0 mm/s) as described by Vicsek et al. (1995)^38^. The larva moves straight ahead and bounces off the outer boundary of the chamber following the law of reflection (angle of incidence = angle of reflection). The larva does not interact with other larvae, i.e. there were no interactions such as collision and attraction/repulsion. Fluid flow was not assumed.

To mimic an experimental chamber with shallow depth having light gradient along x-axis, a two-dimensional rectangular area was assumed which was divided into “light” and “dark” regions at “light–dark boundary” which corresponds to the position (x) at which the light intensity is equal to the larval detection threshold (Supplementary Fig. S4a). We assumed “small” and “large” rectangular areas for the simulation. A two-dimensional “small” rectangular area (7.5 × 2.5 cm), corresponding to experimental chambers used in previous phototaxis studies^10^, and “large” rectangular areas (1.5, 3, 5, 10, 20, 30, and 100 × 0.1 m) assumed the natural open water habitats of corals.

The assumption of a step-down photophobic response in the simulation was described in Supplementary Fig. S4b. When the modeled larva moves from the light to the dark region, it stops moving for a short time period after passing through the light–dark boundary (“time required to stop swimming”, [1] in Supplementary Fig. S4b), remains in a motionless state for a given period (“duration of no swimming period”, [2] in Supplementary Fig. S4b), and then resumes movement along a randomly determined direction with a constant velocity. The position of light–dark boundary and the time required to stop swimming were defined as variables, and the time required to stop swimming was the time value with the mean τ ± 50% fluctuation given by a uniform distribution. The time series change in the mean swimming speed of modeled larvae after passing through the light–dark boundary is shown in Supplementary Fig. S4c. The larvae which pass through the light–dark boundary from the dark side to light side do not respond and continue to swim at a speed of 2.0 mm/s. The period over which larvae stopped swimming ([2] in Supplementary Fig. S4b) did not affect the distribution of larvae in any of the simulations tested, and thus this period was set at a constant value of 4 min. Parameter values including the swimming velocity (2.0 mm/s) and the duration of the no swimming period (4 min.) were based on experimental measurements. Each simulation contains 1000 modeled larvae in a rectangular field.

### Molecular phylogeny of candidate photoreceptor proteins of *Acropora tenuis*

Putative opsin and photolyase/cryptochrome (PLs/CRYs) homologs in *Acropora tenuis* were obtained from the genome sequence data of *Acropora tenuis* (The ReFuGe 2020 Consortium^39^, https://aten.reefgenomics.org/). Amino acid query sequences of previously defined opsins and PLs/CRYs were subjected to BLAST searches (BLASTP and TBLASTN) with an E-value cutoff of 10^–10^. Collected homologs were aligned and trimmed using PRANK^40^ and TrimAl^41^, and then the ML tree was reconstructed using RAxML-HPC version 8^42^ assuming the LG + F + Γ (for opsins) or LG + Γ (for PLs/CRYs) model of protein evolution, which were the best-fit models for the aligned sequences selected by Aminosan^43^, respectively. The ML tree was visualized with FigTree software (https://tree.bio.ed.ac.uk/software/figtree/).

### Ethics

All samples of *Acropora tenuis* were collected under Great Barrier Reef Marine Park Authority permits G12/35236.1 and G17/713999.1 and Okinawa prefectural government permission no. 29-73.

## Supplementary information

Supplementary Information.

Supplementary Movie S1.

Supplementary Movie S2.

Supplementary Movie S3.

Supplementary Movie S4.

Supplementary Movie S5.

## References

[1] Keough MJ, Downes BJ (1982). Recruitment of marine invertebrates: the role of active larval choices and early mortality. Oecologia.

[2] Connell JH (1985). The consequences of variation in initial settlement vs. post-settlement mortality in rocky intertidal communities. J. Exp. Mar. Biol. Ecol..

[3] Grosberg RK, Levitan DR (1992). For adults only? Supply-side ecology and the history of larval biology. Trends Ecol. Evol..

[4] Titlyanov EA, Latypov YY (1991). Light-dependence in scleractinian distribution in the sublittoral zone of South China Sea Islands. Coral Reefs.

[5] Dubinsky Z, Falkowski P, Dubinsky Z, Stambler N (2011). Light as a source of information and energy in zooxanthellate corals. Coral Reefs: An Ecosystem in Transition.

[6] Roberts TE, Bridge TCL, Caley MJ, Madin JS, Baird AH (2019). Resolving the depth zonation paradox in reef-building corals. Ecology.

[7] Suzuki G, Hayashibara T, Shirayama Y, Fukami H (2008). Evidence of species-specific habitat selectivity of *Acropora* corals based on identification of new recruits by two molecular markers. Mar. Ecol. Prog. Ser..

[8] Baird AH, Babcock RC, Mundy CP (2003). Habitat selection by larvae influences the depth distribution of six common coral species. Mar. Ecol. Prog. Ser..

[9] Harrington L, Fabricius K, De’ath G, Negri A (2004). Recognition and selection of settlement substrata determine post-settlement survival in corals. Ecology.

[10] Kawaguti S (1941). On the physiology of reef corals V. Tropisms of coral planulae, considered as a factor of distribution of the reefs. Palao Trop. Biol. Stat. Stud.

[11] Lewis JB (1974). The settlement behaviour of planulae larvae of the hermatypic coral *Favia Fragum*. J. Exp. Mar. Biol. Ecol..

[12] Bassim KM, Sammarco PW (2003). Effects of temperature and ammonium on larval development and survivorship in a scleractinian coral (*Diploria strigosa*). Mar. Biol..

[13] Brooke S, Young CM (2005). Embryogenesis and larval biology of the ahermatypic scleractinian *Oculina varicosa*. Mar. Biol..

[14] Gleason DF, Edmunds PJ, Gates RD (2006). Ultraviolet radiation effects on the behavior and recruitment of larvae from the reef coral *Porites astreoides*. Mar. Biol..

[15] Babcock R, Mundy C (1996). Coral recruitment: consequences of settlement choice for early growth and survivorship in two scleractinians. J. Exp. Mar. Bio. Ecol..

[16] Mundy CN, Babcock RC (1998). Role of light intensity and spectral quality in coral settlement: implications for depth-dependent settlement?. J. Exp. Mar. Bio. Ecol..

[17] Mason B, Beard M, Miller MW (2011). Coral larvae settle at a higher frequency on red surfaces. Coral Reefs.

[18] Strader ME, Davies SW, Matz MV (2015). Differential responses of coral larvae to the colour of ambient light guide them to suitable settlement microhabitat. R. Soc. Open Sci..

[19] Foster T, Gilmour JP (2016). Seeing red: coral larvae are attracted to healthy-looking reefs. Mar. Ecol. Prog. Ser..

[20] Diehn B (1977). Terminology of behavioral responses of motile microorganisms. Photochem. Photobiol..

[21] Nultsch W, Häder DP (1988). Photomovement in motile microorganisms II. Photochem. Photobiol..

[22] Hegemann P, Bruck B (1989). Light-induced stop response in *Chlamydomonas reinhartii*: occurrence and adaptation phenomena. Cell Motil. Cytoskeleton.

[23] Rüffer U, Nultsch W (1990). Flagellar photoresponses of Chlamydomonas cells held on micropipettes: I. Change in flagellar beat frequency. Cell Motil. Cytoskeleton.

[24] Barghigiani C, Colombetti G, Franchini B, Lenci F (1979). Photobehavior of *Euglena gracilis*: action spectrum for the step-down photophobic response of individual cells. Photochem. Photobiol..

[25] Shimmen T (1981). Quantitative studies on step-down photophobic response of *Euglena* in an individual cell. Protoplasma.

[26] Matsunaga S (1998). Discovery of signaling effect of UV-B/C light in the extended UV-A/blue-type action spectra for step-down and step-up photophobic responses in the unicellular flagellate alga *Euglena gracilis*. Protoplasma.

[27] Szmant AM, Meadows MG (2006). Developmental changes in coral larval buoyancy and vertical swimming behavior: implications for dispersal and connectivity. Proc. 10th Int. Coral Reef Symp..

[28] Harrison PL, Wallace CC, Dubinsky Z (1990). Reproduction, dispersal and recruitment of scleractinian corals. Coral Reefs.

[29] Hata T (2017). Coral larvae are poor swimmers and require fine-scale reef structure to settle. Sci. Rep..

[30] Kirk J (2011). Light and Photosynthesis in Aquatic Ecosystems.

[31] Levy O (2007). Light-responsive cryptochromes from a simple multicellular animal, the coral *Acropora millepora*. Science.

[32] Mason B (2012). Evidence for multiple phototransduction pathways in a reef-building coral. PLoS ONE.

[33] Randall CJ (2020). Sexual production of corals for reef restoration in the Anthropocene. Mar. Ecol. Prog. Ser..

[34] Watanabe M (1982). Design and performance of the Okazaki Large Spectrograph for photobiological research. Photochem. Photobiol..

[35] Petersen D, Hatta M, Laterveer M, van Bergen D (2005). Ex situ transportation of coral larvae for research, conservation, and aquaculture. Coral Reefs.

[36] Vermeij MJA, Bak RPM (2002). How are coral populations structured by light?: marine light regimes and the distribution of *Madracis*. Mar. Ecol. Prog. Ser..

[37] R Core Team. *R: a language and environment for statistical computing* (R Foundation for Statistical Computing, 2018).

[38] Vicsek T, Czirók A, Ben-Jacob E, Cohen I, Shochet O (1995). Novel type of phase transition in a system of self-driven particles. Phys. Rev. Lett..

[39] Voolstra CR (2015). The ReFuGe 2020 Consortium-using ‘omics’ approaches to explore the adaptability and resilience of coral holobionts to environmental change. Front. Mar. Sci..

[40] Löytynoja A, Goldman N (2005). An algorithm for progressive multiple alignment of sequences with insertions. Proc. Natl. Acad. Sci. U.S.A..

[41] Capella-Gutiérrez S, Silla-Martínez JM, Gabaldón T (2009). trimAl: a tool for automated alignment trimming in large-scale phylogenetic analyses. Bioinformatics.

[42] Stamatakis A (2014). RAxML version 8: a tool for phylogenetic analysis and post-analysis of large phylogenies. Bioinformatics.

[43] Tanabe AS (2011). Kakusan4 and Aminosan: two programs for comparing nonpartitioned, proportional and separate models for combined molecular phylogenetic analyses of multilocus sequence data. Mol. Ecol. Resour..

