# Supplementary material for: A step-down photophobic response in coral larvae: implications for the light-dependent distribution of the common reef coral, Acropora tenuis
Source: Sci Rep. 2020 Oct 19;10:17680. doi: 10.1038/s41598-020-74649-x (PMC7572417; doi:10.1038/s41598-020-74649-x)
Supplement: Supplementary file 1 — Supplementary Information. [file 41598_2020_74649_MOESM1_ESM.pdf]

Supplementary Information:

A step-down photophobic response in coral larvae: implications for the light-dependent distribution of the common reef coral, *Acropora tenuis*

Yusuke Sakai[1], Kagayaki Kato[2,3,4], Hiroshi Koyama[5,6], Alyson Kuba[7], Hiroki Takahashi[1,6], Toshihiko Fujimori[5,6], Masayuki Hatta[8], Andrew P. Negri[9], Andrew H. Baird[7] and Naoto Ueno[1,6]

1. Division of Morphogenesis, National Institute for Basic Biology, Okazaki, Aichi, Japan.
2. Exploratory Research Center on Life and Living Systems (ExCELLS), National Institutes of Natural Sciences, Okazaki, Aichi, Japan.
3. Department of Imaging Science, Center for Novel Science Initiatives, National institute for Basic Biology, Okazaki, Aichi, Japan.
4. Division of Evolutionary Biology Biodiversity, National Institute for Basic Biology, Okazaki, Aichi, Japan.
5. Division of Embryology, National Institute for Basic Biology, Okazaki, Aichi, Japan.
6. Department of Basic Biology, School of Life Science, SOKENDAI (The Graduate University for Advanced Studies), Okazaki, Aichi, Japan.
7. ARC Centre of Excellence for Coral Reef Studies, James Cook University, Townsville, Queensland, Australia.
8. Department of Biology, Ochanomizu University, Bunkyo-ku, Tokyo, Japan.
9. Australian Institute of Marine Science, Townsville, Queensland, Australia.

Supplementary figures

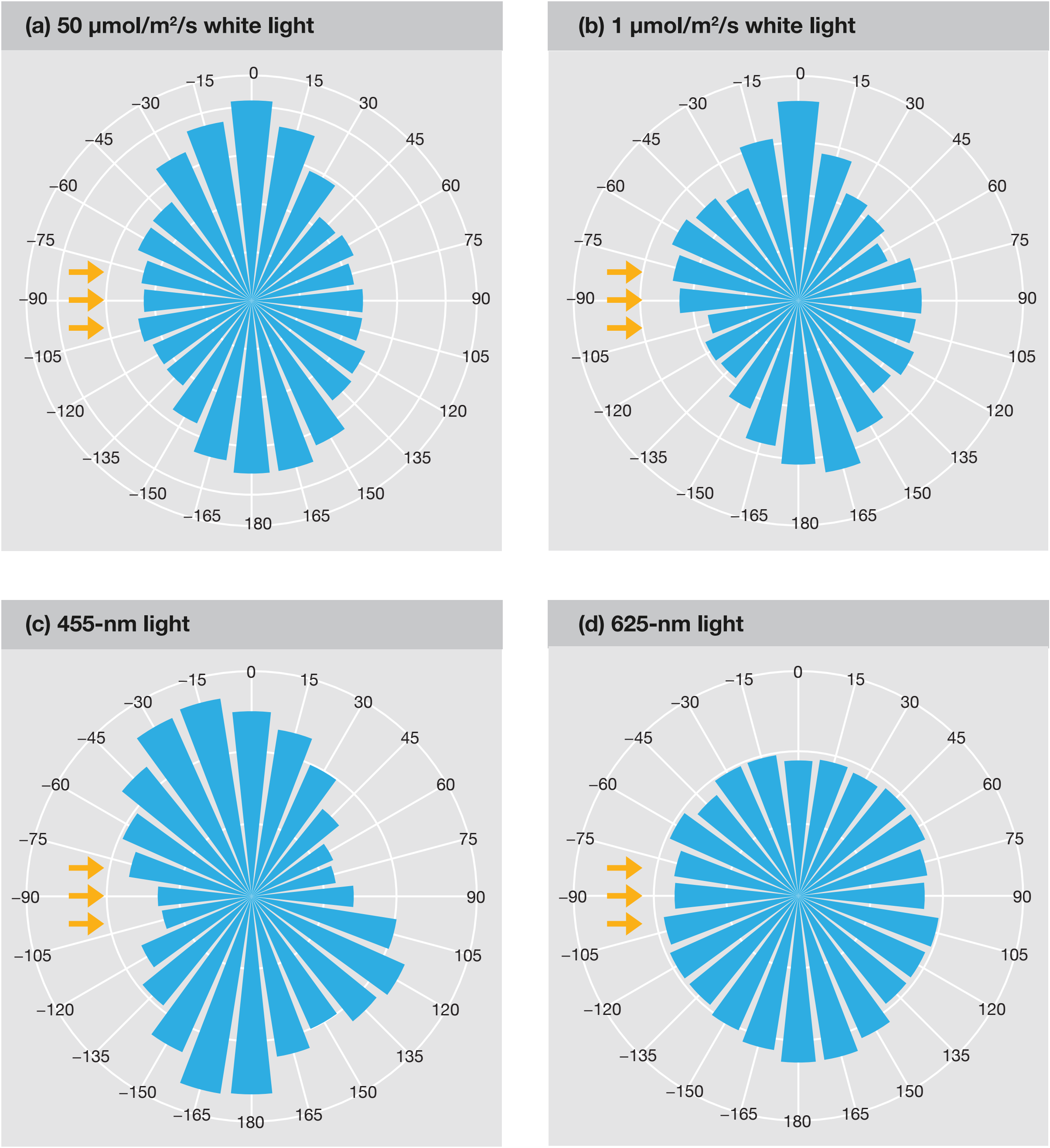

Supplementary Fig. S1. Distributions of the swimming direction of *Acropora tenuis* larvae in the 6.5 cm × 3.5 cm chamber. Each rose diagram summarizes the swimming directions for larval trajectories under (a) 50 μmol/m^2/s white light, (b) 1 μmol/m^2/s white light, (c) 455-nm blue light, and (d) 625-nm red light, respectively. The swimming directions in (b), (c) and (d) were calculated for the interval between 30 and 90 s after light stimuli were switched. The swimming direction is represented as the

degree of rotation in a circle (−180° ~ + 180°). Yellow arrows in the figures represent the direction of the light source (−90°).

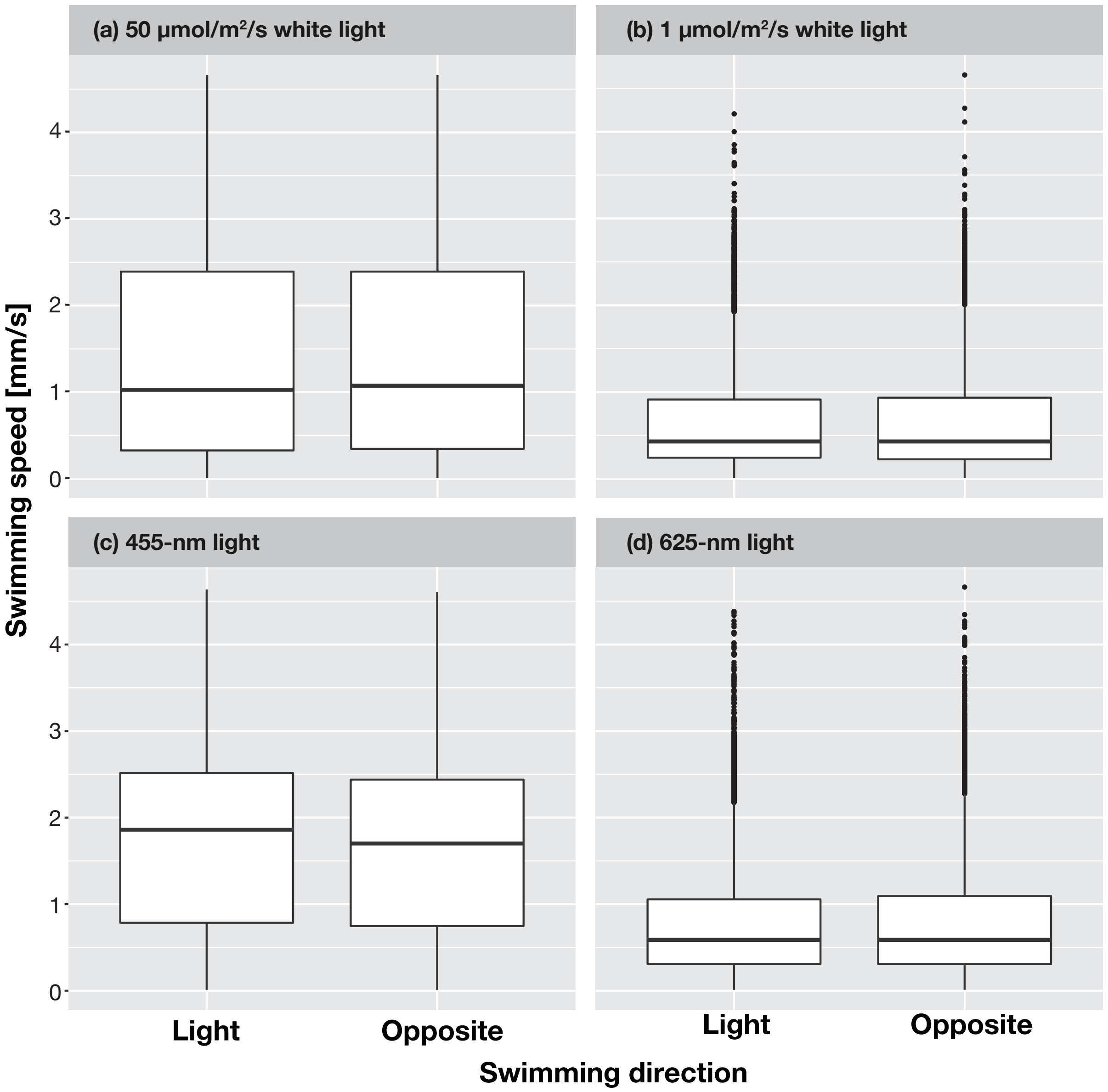

Supplementary Fig. S2. Absolute swimming speed of larvae which move toward the light direction and the opposite direction. The swimming speeds are calculated for four different light conditions shown in Supplementary Fig. S1. The light direction and opposite direction correspond to −60° ~ −120° and +60° ~ +120° in the Supplementary Fig. S1, respectively.

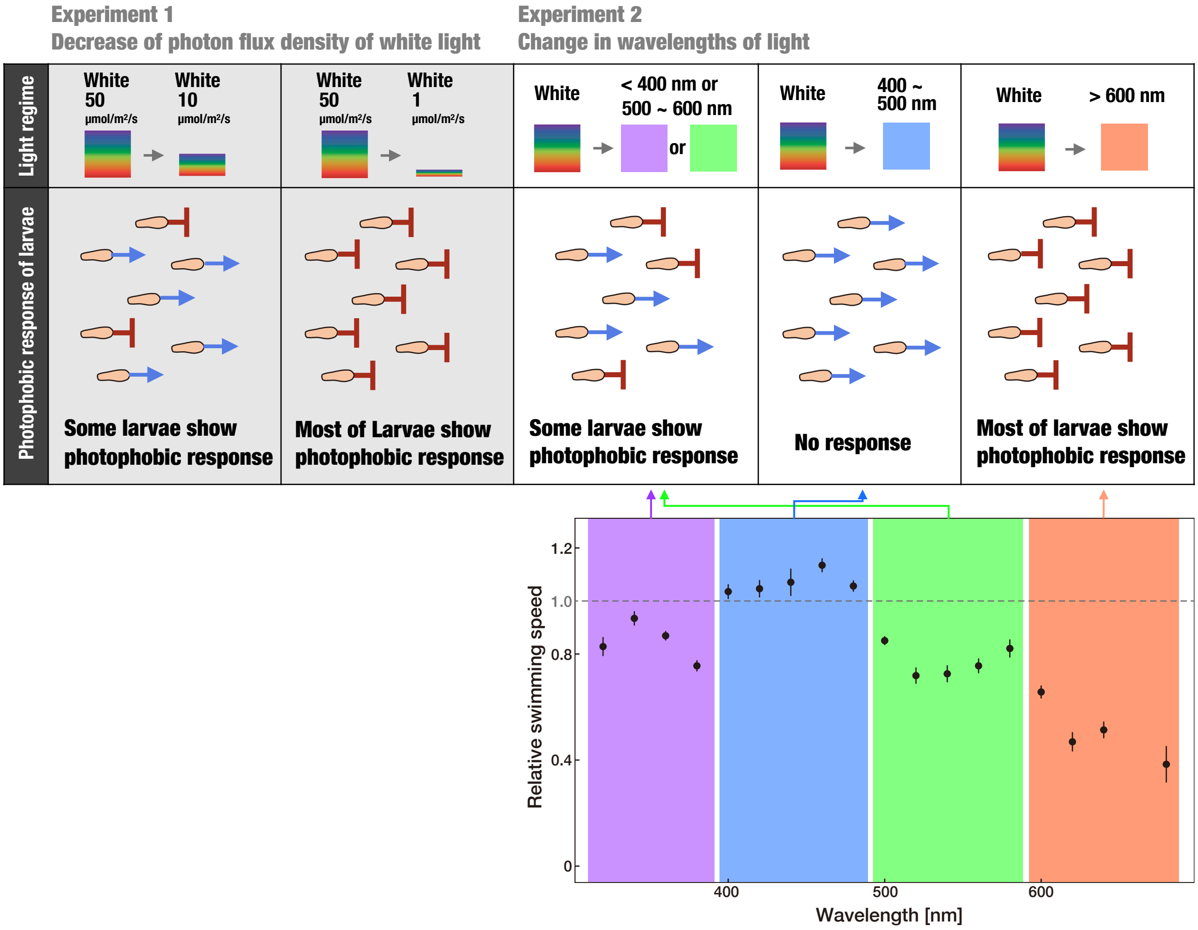

Supplementary Fig. S3. Graphical summary of the result of the differences in the larval photophobic response.

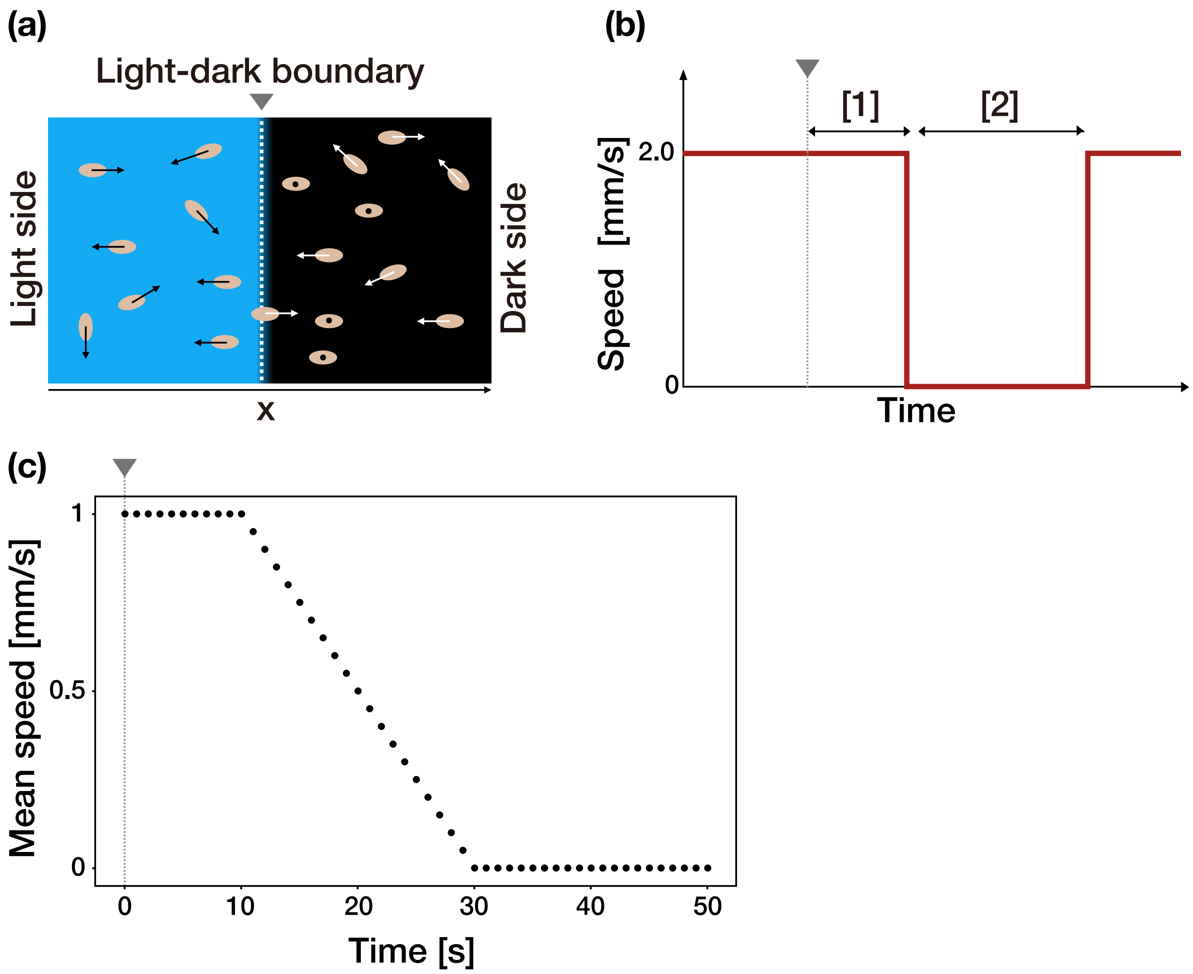

Supplementary Fig. S4. Mathematical simulation of larvae having a step-down photophobic response. (a) A schematic for a swimming field of modeled larvae having step-down photophobic responses, (b) property of swimming behavior of an individual larva in the simulation. The larval behavior in a two-dimensional rectangular field is simulated such that the larvae normally swim at a constant speed of 2.0 mm/s and stop swimming if they pass through the light-dark boundary from light side to dark side (represented by a shaded arrow in the figures). A few seconds are required to stop swimming after passing through the light-dark boundary and the time was defined as “time required to stop swimming” ([1] in the figure b). The stop response lasts for a few minutes (the time was defined as “duration of no swimming period”, [2] in the figure b), and then the larvae start swimming again with the original speed, 2.0 mm/s. There is no behavioral change if the larvae pass through the light-dark boundary from dark side to light side. (c) Mean swimming speed of modeled larvae after passing through the

light-dark boundary. The time required to stop swimming was set to 20 ± 10 s, and the mean swimming speed was predicted under this assumption. The point at time = 0 (represented by a shaded arrow in the figure) is the time point at which the larvae pass through the light-dark boundary from light side to dark side.

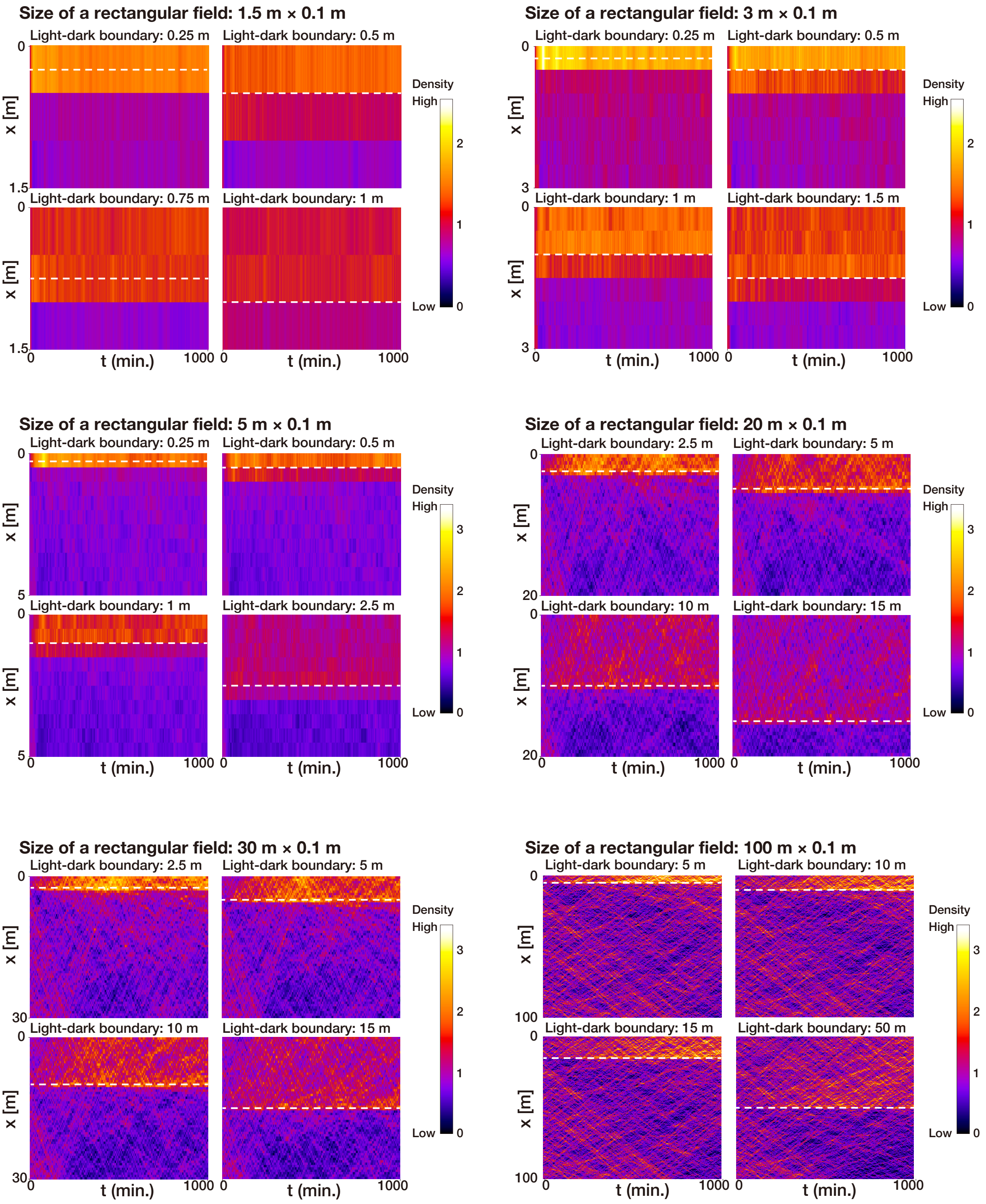

Supplementary Fig. S5. Accumulation of modeled larvae in "large" rectangular fields (1.5, 3, 5, 20, 30, and 100 × 0.1 m). Figures show densities of the larvae along x-axis (x = 0: light side) from 0 to 1,000 minutes. The densities are standardized to a mean density of 1, represented as color brightness, and shown at 0.5 m × 2 minutes resolution in the figures. White dashed lines represent the positions of

the light-dark boundaries. The time required to stop swimming was set to 20 ± 10 s ($\tau = 20$) in the all simulations shown.

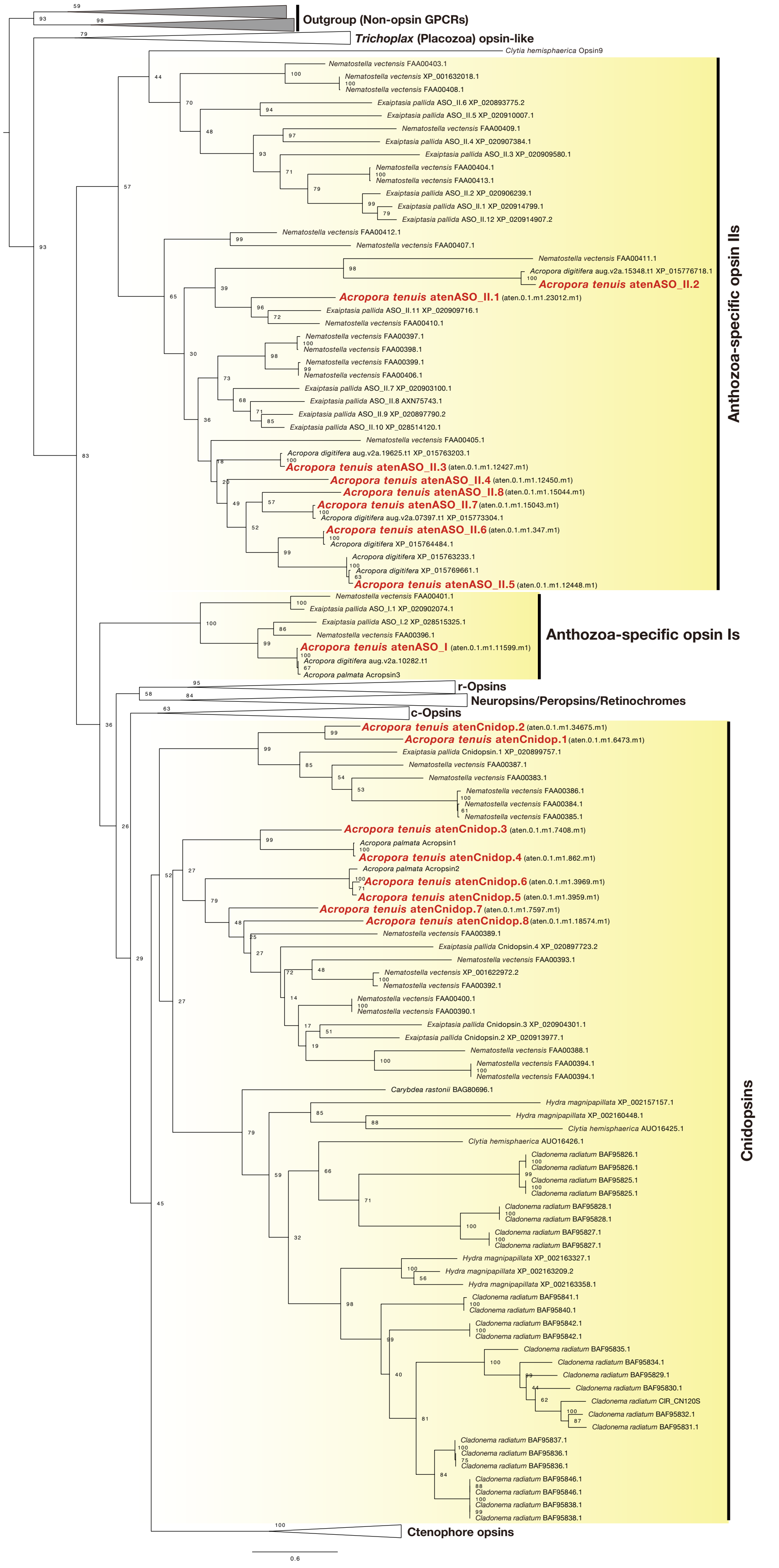

Outgroup (Non-opsin GPCRs)
Trichoplax (Placozoa) opsin-like
Clytia hemisphaerica Opsin9
Nematostella vectensis FAA00403.1
Nematostella vectensis XP_001632018.1
Nematostella vectensis FAA00408.1
Exaiptasia pallida ASO_II.6 XP_020893775.2
Exaiptasia pallida ASO_II.5 XP_020910007.1
Nematostella vectensis FAA00409.1
Exaiptasia pallida ASO_II.4 XP_020907384.1
Exaiptasia pallida ASO_II.3 XP_020909580.1
Nematostella vectensis FAA00404.1
Nematostella vectensis FAA00413.1
Exaiptasia pallida ASO_II.2 XP_020906239.1
Exaiptasia pallida ASO_II.1 XP_020914799.1
Exaiptasia pallida ASO_II.12 XP_020914907.2
Nematostella vectensis FAA00412.1
Nematostella vectensis FAA00407.1
Nematostella vectensis FAA00411.1
Acropora digitifera aug.v2a.15348.t1 XP_015776718.1
Acropora tenuis atenASO_II.2
Acropora tenuis atenASO_II.1 (aten.0.1.m1.23012.m1)
Exaiptasia pallida ASO_II.11 XP_020909716.1
Nematostella vectensis FAA00410.1
Nematostella vectensis FAA00397.1
Nematostella vectensis FAA00398.1
Nematostella vectensis FAA00399.1
Nematostella vectensis FAA00406.1
Exaiptasia pallida ASO_II.7 XP_020903100.1
Exaiptasia pallida ASO_II.8 AXN75743.1
Exaiptasia pallida ASO_II.9 XP_020897790.2
Exaiptasia pallida ASO_II.10 XP_028514120.1
Nematostella vectensis FAA00405.1
Acropora digitifera aug.v2a.19625.t1 XP_015763203.1
Acropora tenuis atenASO_II.3 (aten.0.1.m1.12427.m1)
Acropora tenuis atenASO_II.4 (aten.0.1.m1.12450.m1)
Acropora tenuis atenASO_II.8 (aten.0.1.m1.15044.m1)
Acropora tenuis atenASO_II.7 (aten.0.1.m1.15043.m1)
Acropora digitifera aug.v2a.07397.t1 XP_015773304.1
Acropora tenuis atenASO_II.6 (aten.0.1.m1.347.m1)
Acropora digitifera XP_015764484.1
Acropora digitifera XP_015763233.1
Acropora digitifera XP_015769661.1
Acropora tenuis atenASO_II.5 (aten.0.1.m1.12448.m1)
Anthozoa-specific opsin IIs
Nematostella vectensis FAA00401.1
Exaiptasia pallida ASO_I.1 XP_020902074.1
Exaiptasia pallida ASO_I.2 XP_028515325.1
Nematostella vectensis FAA00396.1
Acropora tenuis atenASO_I (aten.0.1.m1.11599.m1)
Acropora digitifera aug.v2a.10282.t1
Acropora palmata Acropsin3
Anthozoa-specific opsin Is
r-Opsins
Neuropsins/Peropsins/Retinochromes
c-Opsins
Acropora tenuis atenCnidop.2 (aten.0.1.m1.34675.m1)
Acropora tenuis atenCnidop.1 (aten.0.1.m1.6473.m1)
Exaiptasia pallida Cnidopsin.1 XP_020899757.1
Nematostella vectensis FAA00387.1
Nematostella vectensis FAA00383.1
Nematostella vectensis FAA00386.1
Nematostella vectensis FAA00384.1
Nematostella vectensis FAA00385.1
Acropora tenuis atenCnidop.3 (aten.0.1.m1.7408.m1)
Acropora palmata Acropsin1
Acropora tenuis atenCnidop.4 (aten.0.1.m1.862.m1)
Acropora palmata Acropsin2
Acropora tenuis atenCnidop.6 (aten.0.1.m1.3969.m1)
Acropora tenuis atenCnidop.5 (aten.0.1.m1.3959.m1)
Acropora tenuis atenCnidop.7 (aten.0.1.m1.7597.m1)
Acropora tenuis atenCnidop.8 (aten.0.1.m1.18574.m1)
Nematostella vectensis FAA00389.1
Exaiptasia pallida Cnidopsin.4 XP_020897723.2
Nematostella vectensis FAA00393.1
Nematostella vectensis XP_001622972.2
Nematostella vectensis FAA00392.1
Nematostella vectensis FAA00400.1
Nematostella vectensis FAA00390.1
Exaiptasia pallida Cnidopsin.3 XP_020904301.1
Exaiptasia pallida Cnidopsin.2 XP_020913977.1
Nematostella vectensis FAA00388.1
Nematostella vectensis FAA00394.1
Nematostella vectensis FAA00394.1
Carybdea rastonii BAG80696.1
Hydra magnipapillata XP_002157157.1
Hydra magnipapillata XP_002160448.1
Clytia hemisphaerica AUO16425.1
Clytia hemisphaerica AUO16426.1
Cladonema radiatum BAF95826.1
Cladonema radiatum BAF95826.1
Cladonema radiatum BAF95825.1
Cladonema radiatum BAF95825.1
Cladonema radiatum BAF95828.1
Cladonema radiatum BAF95828.1
Cladonema radiatum BAF95827.1
Cladonema radiatum BAF95827.1
Hydra magnipapillata XP_002163327.1
Hydra magnipapillata XP_002163209.2
Hydra magnipapillata XP_002163358.1
Cladonema radiatum BAF95841.1
Cladonema radiatum BAF95840.1
Cladonema radiatum BAF95842.1
Cladonema radiatum BAF95842.1
Cladonema radiatum BAF95835.1
Cladonema radiatum BAF95834.1
Cladonema radiatum BAF95829.1
Cladonema radiatum BAF95830.1
Cladonema radiatum CIR_CN120S
Cladonema radiatum BAF95832.1
Cladonema radiatum BAF95831.1
Cladonema radiatum BAF95837.1
Cladonema radiatum BAF95836.1
Cladonema radiatum BAF95836.1
Cladonema radiatum BAF95846.1
Cladonema radiatum BAF95846.1
Cladonema radiatum BAF95838.1
Cladonema radiatum BAF95838.1
Cnidopsins
Ctenophore opsins
0.6

Supplementary Fig. S6. A maximum likelihood (ML) phylogenetic tree of *Acropora tenuis* opsins. Numbers at nodes represent support values of ML branch estimated by 1,000 bootstrap samplings.

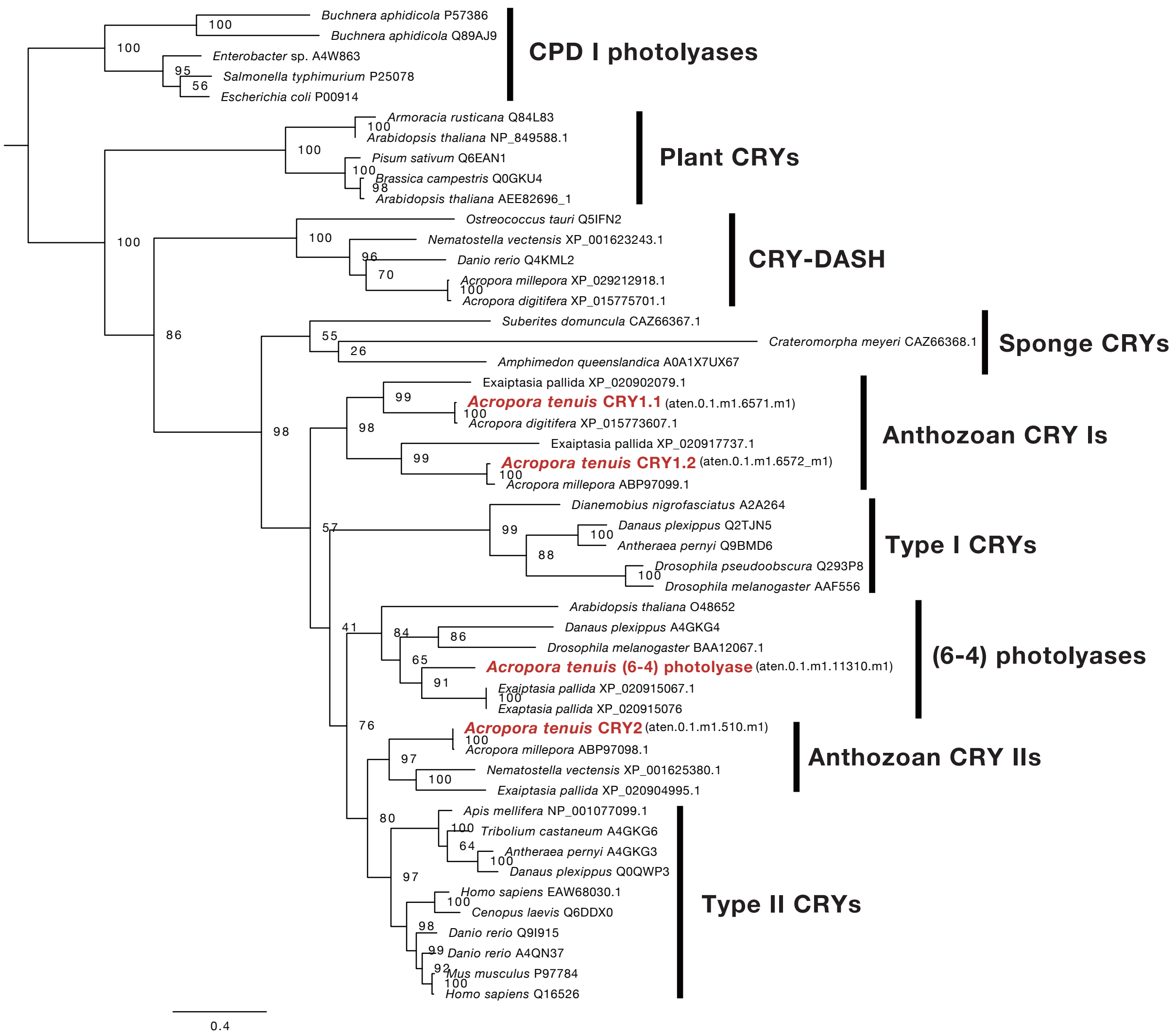

Supplementary Fig. S7. A maximum likelihood (ML) phylogenetic tree of *Acropora tenuis* photolyases and cryptochromes. Numbers at nodes represent support values of ML branch estimated by 1,000 bootstrap samplings.

Supplementary movies

Supplementary Movie S1. Ciliary beating of a swimming larva of *Acropora tenuis* caged in a thin chamber and observed under a differential interference contrast (DIC) transmitted light microscope.

Supplementary Movie S2. A bisected aboral tip part (forward end to the propulsive direction) of a larva of *Acropora tenuis* observed from the cut surface under a DIC transmitted light microscope. The larva shows clockwise rotation as viewed from the cut surface.

Supplementary Movie S3. The larval swimming behavior of *Acropora tenuis* from 20 s before to 240 s after the light attenuation. The light intensity before and after the light attenuation is 50 and 1.1 $\mu mol/m^2/s$, respectively, that corresponds to the light regime shown in Fig. 1d.

Supplementary Movie S4. The swimming behavior from 20 s before to 150 s after the light switching (white light to 455-nm blue light).

Supplementary Movie S5. The swimming behavior from 20 s before to 150 s after the light switching (white light to 625-nm red light).
